# The Breastfeeding Food Security Assessment Scale (BFSAS): Development and Validation Among Breastfeeding Women in Lebanon

**DOI:** 10.1002/fsn3.71355

**Published:** 2025-12-26

**Authors:** Cosette Fakih El Khoury, Joelle Abi Kharma, Petra Nicolas, Rana Rizk, Chadia Haddad, Hala Sacre, Bahia Abdallah, Hala El Mikkawi, Joanne Karam, Pascale Salameh

**Affiliations:** ^1^ Institut National de Santé Publique d’Épidémiologie Clinique et de Toxicologie‐Liban (INSPECT‐LB) Beirut Lebanon; ^2^ Department of Nutrition and Food Science, School of Arts and Sciences Lebanese American University Byblos Lebanon; ^3^ Research Department Psychiatric Hospital of the Cross Jal Eddib Lebanon; ^4^ Faculty of Public Health Lebanese University Fanar Lebanon; ^5^ Alice Ramez Chagoury School of Nursing, LAU‐Lebanese American University Lebanese American University Byblos Lebanon; ^6^ Department of Nutrition and Food Science, School of Arts and Sciences Lebanese American University Beirut Lebanon; ^7^ Faculty of Pharmacy Lebanese University Hadat Lebanon; ^8^ Department of Primary Care and Population Health University of Nicosia Medical School Nicosia Cyprus; ^9^ Gilbert and Rose‐Marie Chagoury School of Medicine Lebanese American University Beirut Lebanon

**Keywords:** breast feeding, developing countries, food insecurity, food security, Lebanon, maternal nutrition, validation study

## Abstract

Food insecurity significantly impacts health, particularly for breastfeeding mothers, who require adequate nutrition for maternal and child health. In Lebanon, economic crises have worsened food insecurity, affecting nearly half of the population. Crrent food insecurity assessment tools do not address the specific needs of breastfeeding women. This study aimed to develop and validate the Breastfeeding Food Security Assessment Scale (BFSAS), a context‐specific tool for assessing food security among breastfeeding mothers in Lebanon, and to examine sociodemographic factors associated with food insecurity within this population. A cross‐sectional survey was conducted from December 2023 to March 2024 among Lebanese mothers who had given birth between 2018 and 2023. Participants were recruited through social media and completed an online questionnaire covering sociodemographic, dietary, mental health, breastfeeding, and food security‐related questions. Data analysis included exploratory and confirmatory factor analyses to validate the BFSAS structure and statistical tests to explore associations with the BFSAS scores. The study included 305 participants, with the BFSAS demonstrating excellent psychometric properties and a two‐factor structure, “quality” and “quantity,” with high internal consistency (Cronbach's *α* = 0.95). Approximately 23% of participants experienced food insecurity during breastfeeding. Older age and higher household income were associated with lower odds of food insecurity, while the number of children in the household increased the likelihood of food insecurity. The BFSAS is a valid, reliable, culturally adapted tool for assessing food insecurity among breastfeeding women in Lebanon. It facilitates targeted interventions to support breastfeeding mothers, especially in regions with lower socioeconomic status.

## Key Messages

- The Breastfeeding Food Security Assessment Scale (BFSAS) is a valid and culturally adapted tool specifically designed to measure food insecurity among BF women in Lebanon.
- The BFSAS demonstrated excellent psychometric properties (Cronbach's *α* = 0.95), ensuring its reliability and validity.
- The study found that about 23% of BF mothers experienced food insecurity during BF.
- Older maternal age and higher household income were associated with lower odds of food insecurity, while the number of children in the household increased the likelihood of food insecurity.

## Graphical abstract

Current food insecurity assessment tools do not address the specific needs of breastfeeding women. We developed and validated the Breastfeeding Food Security Assessment Scale (BFSAS), a context‐specific tool for assessing food security among breastfeeding mothers in Lebanon. The BFSAS is a 7‐item, reliable, culturally adapted, and easy to use tool for assessing food insecurity among breastfeeding women in Lebanon.

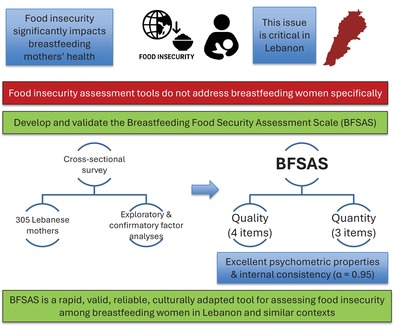

**Abbreviations**
BBQ: Breastfeeding Behavior Questionnaire
BF: breastfeeding
BFSAS: Breastfeeding Food Security Assessment Scale
CFA: confirmatory factor analysis
CFI: Comparative Fit Index
EFA: exploratory factor analysis
EREC: Expected Residual Correlation Direct Change
FIES: Food Insecurity Experience Scale
HFIAS: Household Food Insecurity Access Scale
IPC: Integrated Food Security Phase Classification
IQR: interquartile ranges
KMO: Kaiser–Meyer–Olkin
MEDAS: Mediterranean Diet Adherence Screener
MSA: Measure of Sampling Adequacy
PHQ‐4: Patient Health Questionnaire 4
RMSEA: root mean square error of approximation
SD: standard deviations
SEM: structural equation modeling
SRMR: standardized root mean square residual
TLI: Tucker Lewis Index
WHIIRS: Women's Health Initiative Insomnia Rating Scale

## 1 Introduction

Food insecurity is the inadequate and inconsistent access to sufficient, safe, and nutritious food necessary for healthy growth, development, and an active lifestyle (Food and Agriculture Organization 2024). Globally, 2.3 billion people were estimated to suffer from moderate or severe food insecurity between 2021 and 2023 (UNICEF 2024). In Lebanon, severe food insecurity affected 11.7% of the population, while moderate or severe food insecurity was estimated at 40.1% of the Lebanese population between 2021 and 2023 (UNICEF 2024).

Food insecurity exacerbates malnutrition rates, including stunting and wasting, both of which remain prevalent in Low Middle‐Income Countries (LMICs) (Black et al. 2013; UNICEF 2024). As part of the global mission to eradicate hunger, food insecurity, and malnutrition, the prevalence of undernourishment and the prevalence of moderate or severe food insecurity in the population are used as indicators to measure the progress toward achieving the United Nations Sustainable Development Goal 2 (SDG 2), which aims to achieve zero hunger (United Nations 2024).

Increasing breastfeeding (BF) rates, particularly exclusive BF, is considered a cornerstone in achieving SDG 2. Significant improvements have been made in raising the global exclusive BF rate for infants under 6 months, which increased from 37.1% in 2012 to 48% in 2022. However, the set target of 70% for 2030 is still far from being achieved (UNICEF 2024).

BF, as defined by the World Health Organization (WHO), involves feeding infants human breast milk, either directly from the breast or expressed, to provide optimal nutrition for early development (World Health Organization 2023). WHO guidelines recommend exclusive BF during the first 6 months of life, followed by continued BF with appropriate complementary foods for up to 2 years or beyond (World Health Organization 2023). BF is vital to maternal and child health, offering broad health, economic, and social benefits (Victora et al. 2016). For infants, it boosts immunity, supports growth, and lowers the risks of malnutrition, infections, and childhood obesity while potentially aiding cognitive development (Couto et al. 2020; North et al. 2022). It also protects against common illnesses like respiratory and gastrointestinal infections (Couto et al. 2020). For mothers, BF aids postpartum recovery, reduces cancer risks, and enhances mental well‐being, fostering long‐term social bonds (Krol and Grossmann 2018; Modak et al. 2023; North et al. 2022). A study from Spain, for example, estimated that the Spanish National Health System could save an estimated €5.6 million annually through reduced incidence of infant disease by raising exclusive BF rates by even 1% (Quesada et al. 2020).

The impact of food insecurity on BF practices varies across different populations and study designs. Some studies reported a significant association between food insecurity and shorter BF durations (De Silva et al. 2024; Tomori 2023), while others found no significant impact once confounding factors are adjusted (Guerrero et al. 2024; Wong et al. 2019). Currently validated food insecurity tools do not address the specific experiences of breastfeeding women, particularly in capturing both quantity and quality dimensions of food insecurity. This gap is especially evident in Lebanon, where no breastfeeding‐specific scale has been developed or tested, despite the availability of general tools like the Food Insecurity Experience Scale (FIES) (Cafiero et al. 2018) and the Household Food Insecurity Access Scale (HFIAS) (Coates et al. 2007). The Food and Agriculture Organization (FAO) recommends the use of the Food Insecurity Experience Scale (FIES) (Cafiero et al. 2018) to assess food insecurity at the individual level; however, the scale is not specific or sensitive to BF women. Additionally, the Household Food Insecurity Access Scale (HFIAS) for the measurement of food access is proposed to assess household insecurity (Coates et al. 2007). The lack of tailored tools may limit efforts to accurately identify breastfeeding women experiencing food insecurity and to develop context‐specific public health strategies. Given the multifaceted relationship between BF and food insecurity, accurately assessing food insecurity during BF is crucial, especially given the scarcity of valid tools in countries like Lebanon, where cultural, political, and socioeconomic factors and the economic collapse influence how food insecurity is experienced both at the individual and household levels.

It was hypothesized that food insecurity would be associated with a lower socioeconomic status, a lower education, and a higher number of children.

The primary objective of this article was to develop a culturally adapted, valid, reliable, and easy‐to‐use tool to assess food security among breastfeeding mothers in Lebanon through the validation of the Breastfeeding Food Security Assessment Scale (BFSAS). The secondary objective is to assess, in an economic crisis context, the correlates of the scale regarding sociodemographic characteristics. This study may benefit researchers, healthcare professionals, and policymakers by offering a tool to identify food‐insecure breastfeeding women and inform targeted nutrition support.

## 2 Methods

### 2.1 Study Design and Sampling Strategy

This cross‐sectional study utilized data extracted from a larger survey conducted to assess health and dietary behaviors among Lebanese adult mothers (Abdallah et al. 2025). A non‐probability snowball sampling strategy was employed to recruit participants. Initial participants were recruited through targeted posts and shares in social media groups (Facebook and WhatsApp) related to parenting, nutrition, and maternal health. The survey link was then forwarded through participants' personal networks to recruit additional eligible mothers. This approach facilitated broad geographic coverage across Lebanon, including participants from diverse socioeconomic backgrounds. All responses were collected through a self‐administered online questionnaire using Google Forms, ensuring voluntary, anonymous participation. No incentives were provided.

### 2.2 Participants

Eligible participants were Lebanese women aged 18 and older, residing in Lebanon, outside refugee camps, who had given birth between 2018 and 2023 and were willing to complete an online survey. Women residing in refugee camps, those living outside Lebanon, those unable to access the survey, and women who had not given birth before 2018 were excluded. Women who had breastfed within the past 5 years were included to reflect diverse breastfeeding experiences across varying socioeconomic and geographic backgrounds, particularly in light of Lebanon's shifting political and economic conditions during that time frame. To reduce recall bias, participants were asked to report food insecurity specifically during their breastfeeding period, not at the time of the survey.

### 2.3 Ethical Considerations

The study was approved by the Lebanese American University Institutional Review Board (LAU.SAS.RR2.30/Nov/2023), and all participants provided consent to participate by agreeing to all the related statements available on the survey's initial page.

### 2.4 Data Collection

Data were collected between December 3, 2023, and March 16, 2024. The survey was administered in English and Arabic; mothers could choose their preferred language. The publicly available English versions of some scales were used, and the translation to Arabic was executed following international guidelines. The survey comprised the following questions:

*Sociodemographic, lifestyle, and employment questions* included questions about age, marital status, number of children, educational level, place of residence, area of living, monthly household income, smoking habits, alcohol consumption, intake of dietary supplements, and current occupation.

*Maternity‐related questions* consisted of the number of children and the mothers' occupation during the breastfeeding period.

### 2.5 The Breastfeeding Food Security Assessment Scale (BFSAS) Content Validation

The BFSAS was developed to measure food security experiences, specifically during the BF period. The scale was based on the Food Insecurity Experience Scale (FIES) (Cafiero et al. 2018), recommended by FAO, and the HFIAS (Coates et al. 2007). Both scales are validated and used within the context of Lebanon (Naja et al. 2015; Sheikomar et al. 2021). The FIES is composed of eight questions with simple dichotomous responses of “Yes” or “No,” while the HFIAS is composed of nine questions answered on a 4‐point Likert scale (No, Rarely, Sometimes, and Often), where higher HFIAS scores indicate greater food insecurity levels. The items were reviewed by the expert panel that includes the authors of the article, including three nutritionists, three epidemiologists, and one midwife with extensive experience in breastfeeding promotion. The content review using a Delphi technique led to the choice of 10 items to be included in the newly developed BFSAS scale (90% or more consensus). The questions of the BFSAS were adapted to capture food security challenges relevant to BF mothers in Lebanon. Items from FIES and HFIAS were modified to reflect the local BF context, focusing on household food availability and individual access to nutritious foods. The questionnaire originally used for validation included 10 questions addressing issues such as food sufficiency, variety in food choices, and adjustments families make when food is limited. The answer options ranged from no (1 point) to rarely (1 or 2 times per month during the BF period) (2 points), sometimes (3 to 10 times per month during the BF period) (3 points), and often (more than 10 times per month during the BF period) (4 points). This rating allowed participants to indicate the frequency of these experiences, providing a detailed picture of food security among BF mothers. The BFSAS was translated into Arabic and then revised with a convenience sample of 10 women. Both the English and Arabic versions of the BFSAS were reviewed and approved by the same panel of experts.

The survey also included additional validated tools, such as the PHQ‐4 (for psychological distress), WHIIRS (for sleep quality), MEDAS (for Mediterranean diet adherence), and BBQ (for breastfeeding behavior), which were administered as part of a broader dataset; however, they are not discussed or analyzed in this manuscript.

### 2.6 Sample Size Calculation

The minimum sample size was calculated using two approaches. For the scale validation, as 10 items were to be included at the start of the factor analysis, a ratio of 10 participants per item was applied; thus, a minimum of 100 participants was required.

Moreover, an a priori calculation was performed using G*Power 3.1.9.7 for Windows (Heinrich Heine, Universität Düsseldorf, Düsseldorf, Germany) to allow for multivariable analyses of the scale correlates. Since many dependent variables were quantitative, multiple regressions were used to assess their correlates.

Assuming a calculated effect size of *f* ^2^ = 0.11 (small) and expecting a squared multiple correlation of 0.1 (*R* ^2^ deviation from 0) for the omnibus test of multiple regression, the minimum required sample was *n* = 205, considering an alpha error of 5%, a power of 80%, and allowing 20 predictors to be included in the model. Finally, a minimal sample of 300 participants was targeted to account for potential missing values and conduct factor analysis and multivariable regressions.

### 2.7 Statistical Analysis

Analysis was performed using STATA v.13.1. There were no missing responses in the dataset. The exploratory‐confirmatory (EFA‐CFA) factor analysis technique was used to examine the factor structure of the BFSAS. This combined EFA‐CFA framework represents an established and widely accepted approach for assessing construct validity and is consistent with previously published validation studies (Ephrem et al. 2024; Walton et al. 2023). The main sample was split into two random subsamples in STATA; subsample 1 consisted of one‐third of the participants and was used for EFA (*n* = 102; mean age 32.47 ± 4.37), and subsample 2 consisted of two‐thirds of the participants and was used for CFA (*n* = 203; mean age 32.16 ± 4.70). There were no significant differences between the two subsamples in terms of age (*t* = 1.08, *p* = 0.281, *d* = 0.07), number of children (*z =* 1.132, *p =* 0.2575), and number of children less than 5 years (*z =* 0.724, *p =* 0.4690). The split‐sample approach was intentionally selected to allow independent confirmation of the factor structure and minimize sample‐specific bias (Lorenzo‐Seva 2022).

Two preliminary tests were performed, Bartlett's Test of Sphericity and the Kaiser–Meyer–Olkin (KMO) Test of Sampling Adequacy, to evaluate the suitability of the data for Exploratory Factor Analysis (EFA). A significant Bartlett's Test of Sphericity (*p* < 0.05) supports the factorability of the data (Bartlett 1954; Hair et al. 2010). KMO values range from 0 to 1, with interpretation thresholds commonly as follows: values above 0.60 are acceptable, values between 0.70 and 0.79 are “good,” values between 0.80 and 0.89 are “very good,” and values above 0.90 are “excellent” (Field 2013; Kaiser 1974). The Measure of Sampling Adequacy (MSA) at the item level was also performed. For the MSA, items with values below 0.50 are considered unsuitable for inclusion and may be removed to improve the factor model (Kaiser and Rice 1974). To further refine the factor structure, residual correlations between item pairs (referred to as doublets) were assessed using the Expected Residual Correlation Direct Change (EREC) index. Ideally, EREC values should be close to 0, indicating minimal unexplained correlations. Items that consistently appeared in doublets with high residual correlations were considered for removal to enhance model interpretability (Ferrando et al. 2022). Following these preliminary assessments, EFA was conducted using principal component analysis with direct oblimin rotation, taking into consideration the nature of the questions.

A confirmatory factor analysis (CFA) was conducted thereafter to validate the two‐dimensional model identified in the EFA using structural equation modeling (SEM). Model parameters were estimated using maximum likelihood estimation (MLE). To assess the fit of the model, several goodness‐of‐fit indices were evaluated: the *χ* ^2^/df with values ≤ 5; the Comparative Fit Index (CFI) and the Tucker Lewis Index (TLI) with > 0.90 and > 0.95 for acceptable and excellent fit, respectively (Hu and Bentler 1999; Kline 2023); the Steiger‐Lind Root Mean Square Error of Approximation (RMSEA) with < 0.08 for reasonable fit, along with its corresponding PCLOSE, best if above 0.05 (Steiger 1990); and the standardized root mean square residual (SRMR) with < 0.05 for good fit (Hu and Bentler 1999). Given that multivariate normality was not verified, a non‐parametric bootstrapping procedure was employed (Kline 2023).

Internal consistency was assessed using Cronbach's alpha. Alpha values were interpreted as follows: insufficient if below 0.70, acceptable if between 0.70 and 0.79, good if between 0.80 and 0.89, and excellent if 0.90 or above. Given the limitations of Cronbach's alpha in the presence of multidimensional data, McDonald's Omega was also calculated to provide a more accurate estimate of reliability (McDonald 2013; Sijtsma 2009). The cutoff points for McDonald's Omega (*ω*) are generally similar to those used for Cronbach's alpha (Dunn et al. 2014). In addition, structural validity using Spearman coefficients was conducted.

Descriptive analysis was also conducted to summarize the study variables and to check for out‐of‐range values. Continuous variables were presented as means with standard deviations (SD) or medians with interquartile ranges (IQR) for non‐parametric variables. Categorical variables were represented using frequencies and percentages. The total BFSAS score was calculated using the seven‐item questionnaire with a possible range of 7–28, with higher scores indicating a worse food security status during BF. The score was then dichotomized into two groups: “food secure” for females with a score of 7 and “food insecure” for females with a score greater than 7. The “quality” and the “quantity” subscales were also dichotomized into two groups with thresholds of 4 and 3 for the quality and quantity subscales, respectively. A series of simple logistic regressions were conducted at the bivariate level to examine the association between food insecurity and study variables, including age, number of children, number of children under 5 years, number of rooms in the household (excluding bathrooms), number of individuals living in the household, level of education, household income, employment status, location of the household, and area of residence. Logistic regression was chosen as it is the standard and most appropriate analytical method for modeling binary dependent variables (food security), providing odds ratios that quantify the strength and direction of associations. Variables for inclusion in the multivariate model were selected based on contextual relevance and statistical significance at the bivariate level (*p* < 0.05). This two‐step approach, first screening variables at the bivariate level and then including significant and theoretically relevant predictors in a multivariate model, is a widely used strategy in epidemiological and public health research to control for potential confounding while avoiding model overfitting.

A multiple logistic regression was conducted thereafter, with the BFSAS score serving as the primary dependent variable. The independent variables included in the model were participants' characteristics. The multivariate model allowed adjustment for multiple covariates simultaneously to identify independent predictors of food insecurity, thereby improving the robustness of inference and reducing confounding.

The overall models' goodness‐of‐fit was assessed using Hosmer‐Lemeshow's goodness‐of‐fit statistics. A *p*‐value that exceeds 0.05 suggests no evidence of lack of fit (Hosmer et al. 2013).

Analyses were guided by the COSMIN (COnsensus‐based Standards for the selection of health Measurement INstruments) checklist, which provides internationally accepted standards for the evaluation of psychometric properties in health‐related tools (Terwee et al. 2018).

## 3 Results

### 3.1 Sociodemographic Characteristics

A total of 305 participants completed the survey and were included in the study. Their baseline characteristics are detailed in Table 1. Participants had a mean age of 31.96 years (SD = 4.77). Most were married, held either a university (39.67%) or a postgraduate degree (46.56%) as their highest level of education, and resided in Mount Lebanon (44.59%) and urban areas (70.82%). Among those employed, the main group consisted of individuals working as freelancers, contractors, or in fields outside of healthcare (31.80%). Additionally, 21.31% of the participants reported leaving their jobs to care for newborns, and 20.33% identified as housewives.

**TABLE 1:** Sociodemographic characteristics of study participants (*N* = 305).

Variable	*N* (%)
Marital status	
Married	303 (99.34%)
Divorced	2 (0.66%)
Location of residence	
Beirut	71 (23.28%)
Mount Lebanon	136 (44.59%)
Beqaa	25 (8.20%)
South	42 (13.77%)
North	31 (10.16%)
Area of living	
Urban	216 (70.82%)
Rural	89 (29.18%)
Number of household members	
2	6 (1.97%)
3	127 (41.64%)
4	110 (36.07%)
5	42 (13.77%)
≥ 6	20 (6.56%)
Number of rooms in household (excluding bathrooms)	
1	1 (0.33%)
2	23 (7.54%)
3	82 (26.89%)
4	94 (30.82%)
5	71 (23.28%)
6	21 (6.89%)
≥ 7	13 (4.26%)
Level of education	
≤ High school	42 (13.77%)
University	121 (39.67%)
Postgraduate studies	142 (46.56%)
Employment status	
Employed	174 (57.05%)
Unemployed	131 (42.95%)
Monthly income	
≤ 450$	73 (23.94%)
450$–999$	50 (16.39%)
1000$–2000$	65 (21.31%)
> 2000$	117 (38.36%)

Most households had either 1 (46.89%) or 2 (39.67%) children, with similar distributions for children under 5 years of age, primarily having 1 (70.16%) or 2 (28.20%) young children. Household sizes commonly included 3 (41.64%) or 4 (36.07%) members, with most homes featuring 3 (26.89%), 4 (30.82%), or 5 (23.28%) rooms, excluding bathrooms. Monthly household income was most commonly reported as over $2000 (38.36%), with similar proportions in the $1000–$2000 categories and below $450 (21.31%).

### 3.2 Breastfeeding Indicators

Almost all females (96%) reported having ever breastfed, and 85% reported exclusively breastfeeding their infants. A total of 66.23% initiated breastfeeding within 1 h of delivery, while 32.79% introduced formula to their infants before 6 months of age. Those who did not breastfeed cited medical reasons or personal choice as the main reasons for not doing so.

Nearly half of the women reported working during BF, with 54% indicating that their maternity leave lasted 70 days. Among those who reported BF, the median duration was 12 months [IQR: 6–18 months]. For those who reported exclusive BF, the median duration was 6 months [IQR: 4–7 months].

### 3.3 Exploratory Factor Analysis

None of the scale items were removed initially due to low Measure of Sampling Adequacy (MSA). However, two items (Q4 and Q6) were removed due to low communalities (< 0.3). Additionally, item Q9 was removed due to its appearance in multiple high residual correlations or “doublets,” specifically with BFSASQ2, BFSASQ7, and BFSASQ10, as identified through the EREC index. Following these adjustments, the factor analysis was conducted on the remaining seven items. The Bartlett's Test of Sphericity was significant, *χ* ^2^(21) = 1234.964, *p* < 0.001, indicating that the correlations among items were sufficient for factor analysis. The overall KMO value was 0.834, which is considered very good, suggesting high sampling adequacy for the analysis. Inspection of the scree plot of the parallel analysis, including simulated data, suggests that the BFSAS can have up to two factors.

### 3.4 Confirmatory Factor Analysis

A confirmatory factor analysis was conducted to verify the two‐dimensional structure of the BFSAS. The hypothesized model comprised two latent factors: Factor 1, representing the “quality” subscale, and Factor 2, representing the “quantity” subscale. Each factor was measured by a set of observed variables. Specifically, items 1, 2, 3, and 5 were hypothesized to load on the “quality” subscale, while items 7, 8, and 10 were hypothesized to load on the “quantity” subscale.

**FIGURE 1:**
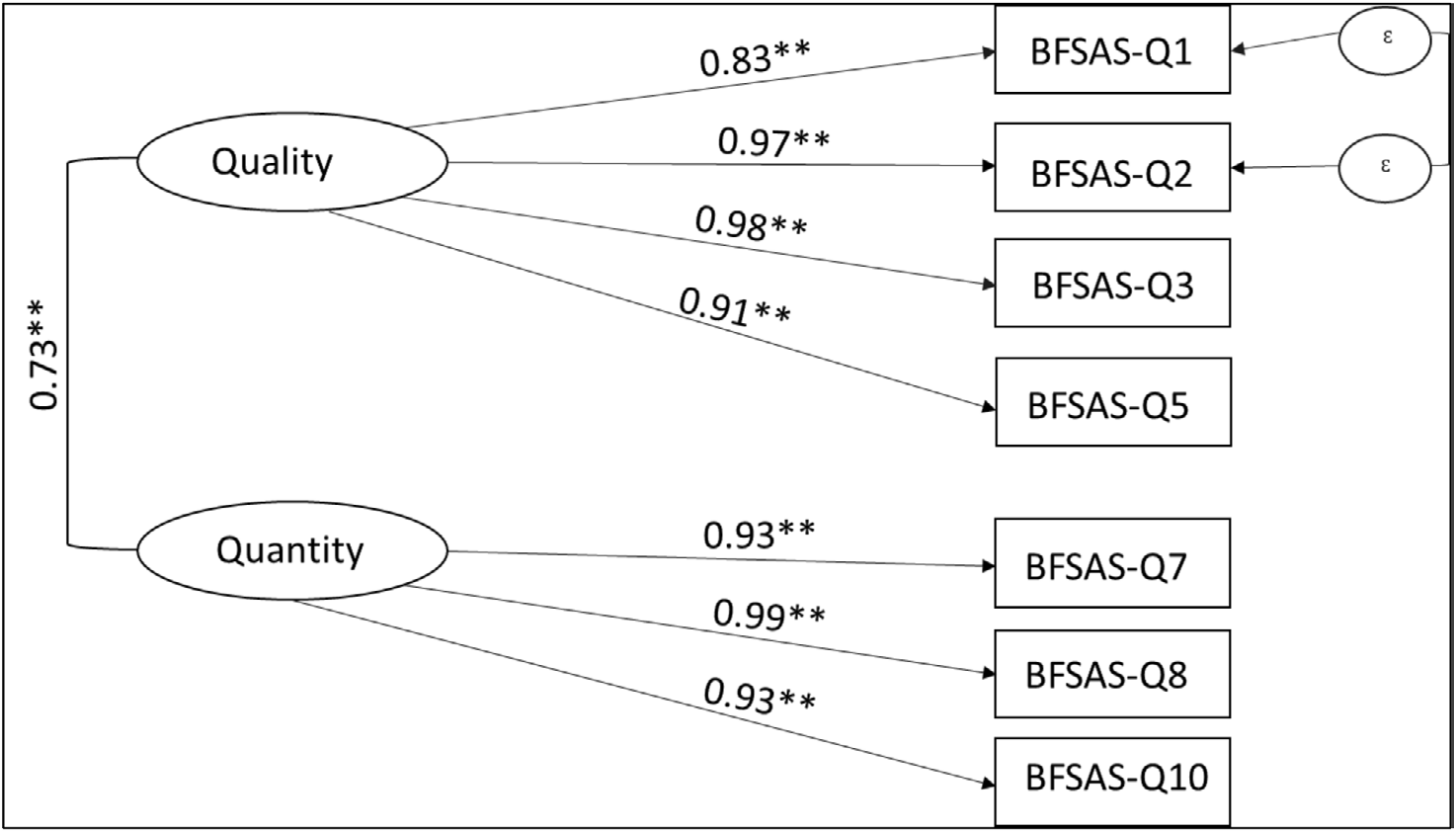
Structural equation model with standardized path coefficients of BFSAS dimensions. **p < 0.01.

The factor loading coefficients supported the hypothesized two‐dimensional model, with factor loadings ranging from 0.86 to 0.98 for the quality subscale (*p* < 0.001) and from 0.94 to 0.99 for the quantity subscale (*p* < 0.001). Goodness‐of‐fit indices indicated an acceptable model fit, with *χ* ^2^/df = 37.94/13 = 2.92 (< 5), CFI = 0.98, TLI = 0.98, and SRMR = 0.011. However, the RMSEA was 0.097, with a PCLOSE of 0.16, indicating that model fit could be further improved. To address this, modification indices were generated, resulting in the re‐specification of the model, allowing the residuals of two conceptually similar items (BFSASQ1 and BFSASQ2) to correlate. BFSASQ1 asked, “During the BF period, did you worry that your household would not have enough food because of a lack of money or other resources?” and Q2 asked, “During the BF period, were you or any household member unable to eat healthy and nutritious food because of a lack of money or other resources?” Allowing these residuals to correlate improved the model's fit significantly, resulting in an RMSEA of 0.031, with a PCLOSE of 0.671. Other goodness‐of‐fit indices for the re‐specified model also indicated excellent fit: *χ* ^2^/df = 14.41/12 = 1.2, CFI = 0.99, TLI = 0.99, and SRMR = 0.008. Figure 1 illustrates the structural equation model with standardized path coefficients of BFSAS dimensions, and Table 2 displays the specific items associated with each subscale.

**TABLE 2:** Final structure of the Breastfeeding Food Security Assessment Scale (BFSAS).

Subscale 1: Quality
Q1. During the breastfeeding period, did you worry that your household would not have enough food because of a lack of money or other resources? لا يكون لأسرتك ما يكفي من الطعام بسبب نقص المال أو الموارد الأخرى؟ خلال فترة الرضاعة، هل قلقتِ من أن
Q2. During the breastfeeding period, were you or any household member unable to eat healthy and nutritious food because of a lack of money or other resources? خلال فترة الرضاعة، هل كنت أنت أو أي فرد من أفراد أسرتك غير قادرين على تناول طعام صحي ومغذي بسبب نقص المال أو الموارد الأخرى؟
Q3. During the breastfeeding period, did you or any household member have to eat a limited variety of food because of a lack of money or other resources? خلال فترة الرضاعة، هل اضطررت أنت أو أي فرد من أفراد أسرتك إلى تناول أنواع محدودة من الطعام بسبب نقص المال أو الموارد الأخرى؟
Q5. During the breastfeeding period, did you or any household member have to eat some foods that you really did not want to eat because of a lack of money or other resources to obtain other types of food? خلال فترة الرضاعة، هل اضطررت أنت أو أي فرد من أفراد أسرتك إلى تناول بعض الأطعمة التي لم ترغبوا في تناولها بسبب نقص المال أو الموارد الأخرى؟

Subscale 2: Quantity
Q7. During the breastfeeding period, was there ever no food to eat of any kind in your household because of a lack of money or other resources to get food? خلال فترة الرضاعة، هل أدى نقص المال أو الموارد الأخرى الى عدم تواجد أي نوع طعام في منزلك؟ (غياب كلي للطعام)؟
Q8. During the breastfeeding period, was there a time when you or any household member were hungry because there was not enough money or other resources to get food? كان هناك وقت شعرت أنت أو أي فرد من أفراد أسرتك بالجوع بسبب عدم وجود ما يكفي من المال أو الموارد الأخرى للحصول على الطعام؟ خلال فترة الرضاعة، هل
Q10. During the breastfeeding period, did you or any household member go to sleep at night hungry because there was not enough money or other resources to get food? خلال فترة الرضاعة، هل ذهبت أنتِ أو أي فرد من أفراد أسرتك إلى النوم و أنتم جائعون بسبب عدم وجود ما يكفي من المال أو الموارد الأخرى للحصول على الطعام؟

### 3.5 Structural Validity

The correlation between the two factors in the confirmatory model was also calculated. The “quality” subscale was positively correlated with the “quantity” subscale (rho = 0.65, *p* < 0.001). The BFSAS scale also demonstrated great structural validity, with Factor 1 having an excellent Spearman correlation with the total scale (rho = 0.995; *p* < 0.001) and Factor 2 a very good correlation (rho = 0.669; *p* < 0.001).

### 3.6 Internal Consistency and Reliability

The seven‐item BFSAS analysis revealed a McDonald's *ω* of 0.95 and a Cronbach's *α* of 0.95, indicating excellent internal consistency for the overall scale. For the two subscales, item analyses showed that subscale 1 (quality) had a Cronbach's *α* of 0.96 and McDonald's *ω* of 0.96, while subscale 2 (quantity) had a Cronbach's *α* of 0.96 and McDonald's *ω* of 0.97. Additionally, item analysis indicated that removing any individual item from the scale did not result in a significant improvement in Cronbach's *α*.

### 3.7 Associations of BFSAS and Participants' Characteristics

A subset of 22.30% of the females was identified as food insecure. The median BFSAS total score for those identified as food insecure was 13 [IQR: 9–18]. For the quality subscale, a similar proportion of 22.30% had at least one positive response to the items on the subscale, with a median score of 9 [IQR: 6–13]. In contrast, only 9.18% of participants had at least one positive response on the quantity subscale, with a median score of 6 [IQR: 4–8].

Factors associated with the risk of food insecurity were age, number of children in the household, number of rooms in the household, household location, level of education, employment status, and monthly income, as shown in Table 3. Specifically, females of older age, having a higher number of rooms in the household, those with postgraduate education, and monthly incomes higher than $450 had lower odds of being identified as food insecure compared to those of younger age, residing in households with a lower number of rooms, having a high school education or lower (*p* < 0.001), and incomes ≤ $450 (*p* = 0.029 for $450 and $999 and *p* < 0.001 for both between $1000 and $2000 and above $2000, respectively). On the contrary, mothers who were not employed, those living in the Beqaa, South, and North regions, and those with a higher number of children in the household had higher odds of being identified as food insecure compared to those who were employed (*p* = 0.007), living in Mount Lebanon (*p* = 0.003, *p* = 0.033, and *p* = 0.019 for the Beqaa, South, and North regions, respectively), and had fewer children in the household (*p* = 0.011).

**TABLE 3:** Bivariate and multivariate logistic regression analyses exploring associations between BFSAS and participants' characteristics.

Variable	Bivariate analysis		Multivariate logistic analysis	
	Odds ratio (95% CI)	*p*	Odds ratio (95% CI)	*p*
Age^a^	0.90 (0.85; 0.96)	0.001*	0.88 (0.81; 0.95)	0.004
Number of children^a^	1.49 (1.09; 2.03)	0.011*	2.01 (1.29; 3.14)	0.002
Number of children < 5 years^a^	1.32 (0.79; 2.20)	0.277	—	—
Number of rooms in household^a^	0.73 (0.58; 0.92)	0.010*	0.91 (0.71; 1.17)	0.473
Number of individuals in household^a^	1.04 (0.98; 1.11)	0.198	—	—
Level of education^b^				
University	0.05 (0.24; 1.03)	0.063*	1.13 (0.47; 2.74)	0.781
Postgraduate studies	0.18 (0.08; 0.40)	< 0.001*	1.03 (0.37; 2.89)	0.953
Household location^c^				
Bekaa	4.11 (1.6; 10.46)	0.003*	0.97 (0.31; 3.11)	0.971
South	2.46 (1.08; 5.62)	0.033*	0.49 (0.17; 1.40)	0.187
North	2.93 (1.19; 7.18)	0.019*	1.13 (0.37; 3.38)	0.828
Beirut	1.94 (0.93; 4.02)	0.075	1.04 (0.43; 2.51)	0.926
Area of residence^d^				
Rural	1.44 (0.81; 2.56)	0.210	—	—
Occupation^e^				
Unemployed	2.11 (1.23; 3.66)	0.007*	0.81 (0.40; 1.63)	0.562
Income^f^				
450$–999$	0.43 (0.20; 0.91)	0.029	0.52 (0.23; 1.19)	0.125
1000$–2000$	0.15 (0.06; 0.34)	< 0.001	0.16 (0.06; 0.42)	< 0.001
> 2000$	0.041 (0.01; 0.11)	< 0.001	0.05 (0.01; 0.16)	< 0.001

^a^ Continuous variable.

^b^ Reference group “≤ high school.”

^c^ Reference group “Mount Lebanon.”

^d^ Reference group “urban.”

^e^ Reference group “employed.”

^f^ Reference group “≤ 450$.”

### 3.8 Multiple Logistic Regression of Food Insecurity and Potential Variables

Age, number of children in the household, and income were identified as independent predictors of food insecurity. Specifically, older age (*p* = 0.004) and higher household income, between $1000 and $2000 (*p* < 0.001) or above $2000 (*p* < 0.001), were associated with significantly lower odds of being identified as food insecure. In contrast, females with a higher number of children in the household had higher odds of being food insecure (*p* = 0.002). The Hosmer‐Lemeshow goodness‐of‐fit test indicated that both models fit the data well, with *p* = 0.7365, suggesting a good model fit.

## 4 Discussion

BF is a cornerstone for food security and health. The 2023 *Lancet Breastfeeding Series* emphasizes that fostering an enabling environment for BF requires coordinated efforts and investment across various domains (Pérez‐Escamilla et al. 2023). In this context, assessing food security during BF is crucial and necessitates using valid and reliable tools to ensure a comprehensive understanding and tailored interventions. The newly developed BFSAS is derived from two validated tools, one addressing food security at the individual level (Cafiero et al. 2018) and the other at the household level (Coates et al. 2007), and aims to provide a robust method for evaluating food security among BF women. The choice of the tool name reflects the importance of BF in achieving food security (Tomori 2023) while acknowledging the potential bidirectional relationship where food insecurity can impact BF rates and practices (De Silva et al. 2024). The decision to recruit women who had breastfed in the past 5 years, rather than only current breastfeeding mothers, allowed for a more diverse and representative validation sample, encompassing various economic and social contexts. While recall bias is a limitation in retrospective reporting, the BFSAS was explicitly framed to assess food insecurity during the breastfeeding period, not the last month, thereby maintaining focus on relevant timeframes. This approach mirrors how perinatal tools often capture retrospective experiences. In contrast to existing instruments like HFIAS and FIES, which do not target breastfeeding‐specific vulnerabilities, the BFSAS provides a culturally grounded, individual‐level assessment better suited to maternal‐child nutrition programming in crisis‐affected LMICs.

The present study aimed to validate the BFSAS to assess food security among BF women in Lebanon for potential use in English and Arabic. Thus, we developed and validated the BFSAS among Lebanese mothers and assessed, in an economic crisis context, the correlates of the scale related to sociodemographic characteristics. The developed scale had excellent psychometric properties, explaining 88% of the latent variable variance, with appropriate validity and reliability. The study identified a seven‐item scale with two primary factors in the BFSAS: “quality” and “quantity,” and demonstrated high internal consistency and reliability. Distinguishing between quality and quantity dimensions is useful in breastfeeding populations, given that maternal diet adequacy may influence maternal and infant health (Carretero‐Krug et al. 2024; Petersohn et al. 2024). Our study showed a comparable internal consistency (Cronbach's *α* of 0.95) to that reported in the validation of the Arabic version of the HFIAS (Cronbach's *α* of 0.91) (Naja et al. 2015).

The identification of the two factors “quality” and “quantity” is rooted in the tools on which the BFSAS was based, the HFIAS (Coates et al. 2007) and particularly the FIES (Cafiero et al. 2018), which identified the same factors. Both of these scales, which were used as the foundation for the development of the BFSAS, have been validated in the Lebanese population (Naja et al. 2015; Sheikomar et al. 2021) and provide a strong basis, enhancing BFSAS credibility as a tool adapted for BF women in Lebanon. The two factors, “quality” and “quantity,” also reflect the interrelated perspectives of food security pertinent to “enough” and “nutritious” food used in the definition of food insecurity (UNICEF 2024).

The sample of Lebanese mothers included a majority of young, well‐educated participants, with distribution from all Lebanese regions and similar socioeconomic status levels. Nevertheless, while most mothers did not have any food security issues (77%), around 23% declared at least some food insecurity concerns, showing a relatively homogeneous sample from that aspect. According to the latest Integrated Food Security Phase Classification (IPC) report from May 2024, poverty in Lebanon has increased further over the past decade, now affecting around 44% of the population, with 23% of the population being food insecure (Integrated Food Security Phase Classification 2024). Compared with national food security data, this study's sample represents a relatively more stable socioeconomic group, given that participants included only Lebanese residents outside refugee camps. Accordingly, our sample does not capture the more vulnerable communities residing in Lebanon.

The geographical residence of the participants in this study should also be addressed, as participants were primarily from urban areas with higher education levels and incomes, and thus, they may experience lower food insecurity compared with the broader Lebanese population and BF women of lower socioeconomic status. Similarly, other studies in Lebanon have shown discrepancies across geographical regions, where Akkar and the Beqaa were also identified to have a higher prevalence of low food consumption scores, 72.9% and 83.2%, respectively, as compared with other regions such as Mount Lebanon (38.8%) (Hoteit et al. 2021). Nevertheless, as expected, the BFSAS was associated with the socioeconomic and demographic variables: higher crowding index, lower income, higher number of children, lower education level, and lower age. This result is consistent with previous national findings from Lebanon showing that lower SES, higher household density, and lower education are strong predictors of food insecurity among women (Jomaa et al. 2020), reinforcing the construct validity of the BFSAS. Similarly, other authors have reported correlations of food insecurity to low educational attainment, unemployment, and crowding from a national study of Lebanese women (Jomaa et al. 2020). Associations with socioeconomic variables confirm the validity of the newly developed scale and indicate its robustness for use in many settings.

Although the association between food insecurity and BF remains inconclusive and may vary across settings (De Silva et al. 2024; Guerrero et al. 2024; Tomori 2023; Wong et al. 2019), food insecurity can have an impact on maternal diets. For instance, a study in Beirut demonstrated that mothers from food‐insecure households had a high risk of dietary inadequacy and obesity (Jomaa et al. 2017). A more recent study also showed inverse associations between household food insecurity and a Healthy Eating Index (HEI) among Lebanese mothers (Jomaa et al. 2020). These findings also validate the relevance of the newly developed tool, indicating a need to capture food security accurately among BF mothers specifically.

A noteworthy result relates to the high rates of self‐reported BF (ever breastfed 96%) and exclusive BF (85%) among this sample as compared to national data, indicating that 70% of children in Lebanon are not exclusively breastfed (Lebanon Nutrition Sector 2022). Among participants of this study, 21.31% reported leaving their jobs to care for their infants, while 20.33% identified as housewives. Maternal employment has been consistently identified among the primary barriers to BF among Lebanese women (Nabulsi 2011), combined with national work policies that fail to adequately support BF mothers, such as providing sufficient time to breastfeed during working hours (Abou Jaoude et al. 2021). Accordingly, the higher BF rates observed in this study may be explained by the fact that nearly half of the sample was not employed during the BF period.

### 4.1 Strengths and Limitations

The main strength of the scale is that it is designed based on two validated tools and that it has been adapted specifically for BF women. Given that the BFSAS is a short scale of seven items, it provides a practical tool for screening food security issues related to quality and quantity among BF mothers, possibly improving response rates. Accordingly, future research is expected to better inform the level of food insecurity among BF mothers and its potential impact on nutritional and growth parameters of both mothers and infants. This study has, however, some limitations related to the characteristics of the participants; a potential selection bias likely influenced the homogeneity of the sample, limiting generalizability to more vulnerable breastfeeding populations, such as refugees or low‐income mothers in rural areas. In addition, relying on self‐declaration might be associated with information bias, while it is expected to drive the associations toward the null and underestimate their value and statistical significance. Moreover, the psychometric validation did not include a test–retest assessment, which would be important to evaluate the scale's stability over time. The lack of external validation with nutrition or health outcomes limits our ability to assess predictive validity. Further studies are suggested to confirm the value of this work, extend it to other populations, and add to it some complementary validation processes, such as a test–retest reliability measure.

## 5 Conclusion

In conclusion, the BFSAS is a reliable and valid seven‐item instrument for assessing food security among BF women in Lebanon, demonstrating excellent psychometric properties, i.e., internal consistency and construct, structural, convergent, and known‐groups validity. This scale is timely, given the rising food insecurity in Lebanon and its potential impact on BF and other health aspects for both mother and baby. Future studies should assess its validity in similar settings to expand its applicability and support its use in addressing food security challenges globally.

## Author Contributions

C.H., H.S., R.R., C.F.E.K., B.A., J.K., and P.S. designed the research; R.R., C.F.E.K., B.A., and J.K. collected the data; J.A.K. and C.F.E.K., analyzed the data. CH, HS, RR, C.F.E.K., B.A., R.F., J.K., J.A.K., H.E.M., and P.S. interpreted the results; C.F.E.K., J.A.K., and P.N. wrote the paper. H.S., R.R., B.A., C.H., P.S., H.E.M., and J.K. reviewed the manuscript. All authors read and approved the final manuscript.

## Funding

The authors have nothing to report.

## Ethics Statement

The study received ethical approval from the Lebanese American University‐ Institutional Review Board (LAU‐IRB) (LAU.SAS.RR2.30/Nov/2023). Before completing the questionnaire, all participants provided their consent by clicking on the consent button “I agree to participate in this study” on the first page of the Google Form.

## Consent

The authors have nothing to report.

## Conflicts of Interest

The authors declare no conflicts of interest.

## Data Availability

The datasets generated during the current study are available from the corresponding author on reasonable request.
